# The Great Irish Famine: Identifying Starvation in the Tissues of Victims Using Stable Isotope Analysis of Bone and Incremental Dentine Collagen

**DOI:** 10.1371/journal.pone.0160065

**Published:** 2016-08-10

**Authors:** Julia Beaumont, Janet Montgomery

**Affiliations:** 1 School of Archaeological Sciences, University of Bradford, Bradford, United Kingdom; 2 Department of Archaeology, Durham University, Durham, United Kingdom; Museo Nazionale Preistorico Etnografico 'L. Pigorini', ITALY

## Abstract

The major components of human diet both past and present may be estimated by measuring the carbon and nitrogen isotope ratios (δ^13^C and δ^15^N) of the collagenous proteins in bone and tooth dentine. However, the results from these two tissues differ substantially: bone collagen records a multi-year average whilst primary dentine records and retains time-bound isotope ratios deriving from the period of tooth development. Recent studies harnessing a sub-annual temporal sampling resolution have shed new light on the individual dietary histories of our ancestors by identifying unexpected radical short-term dietary changes, the duration of breastfeeding and migration where dietary change occurs, and by raising questions regarding factors other than diet that may impact on δ^13^C and δ^15^N values. Here we show that the dentine δ^13^C and δ^15^N profiles of workhouse inmates dating from the Great Irish Famine of the 19th century not only record the expected dietary change from C_3_ potatoes to C_4_ maize, but when used together they also document prolonged nutritional and other physiological stress resulting from insufficient sustenance. In the adults, the influence of the maize-based diet is seen in the δ^13^C difference between dentine (formed in childhood) and rib (representing an average from the last few years of life). The demonstrated effects of stress on the δ^13^C and δ^15^N values will have an impact on the interpretations of diet in past populations even in slow-turnover tissues such as compact bone. This technique also has applicability in the investigation of modern children subject to nutritional distress where hair and nails are unavailable or do not record an adequate period of time.

## Introduction

Famine was a regular occurrence in post-medieval, pre-Industrial Revolution Europe [1,2]. Where the population was mainly rural, any factor which reduced the quantity of food crops, whether climate, military action, or pestilence, would have a devastating effect on the prices of food and the people who relied on the crops for their calories [3]. In the Great Famine of 1845–46, an attempt was made by Sir Robert Peel to provide relief for the Irish by the importation of maize (‘Indian meal’) from America. This unfamiliar food was unpopular, difficult to process and cook: its yellow colour and effects on the intestines of the starving Irish led to it being renamed ‘Peel’s Brimstone’[4]. The δ^13^C values of potatoes, a C_3_ plant and the main food crop in Ireland prior to the Famine, and maize, a C_4_ plant which as a group are largely absent from Ireland at this time, are measurably different and this isotopic shift offers the opportunity to investigate dietary change in a population suffering from documented under-nutrition.

**Fig 1:**
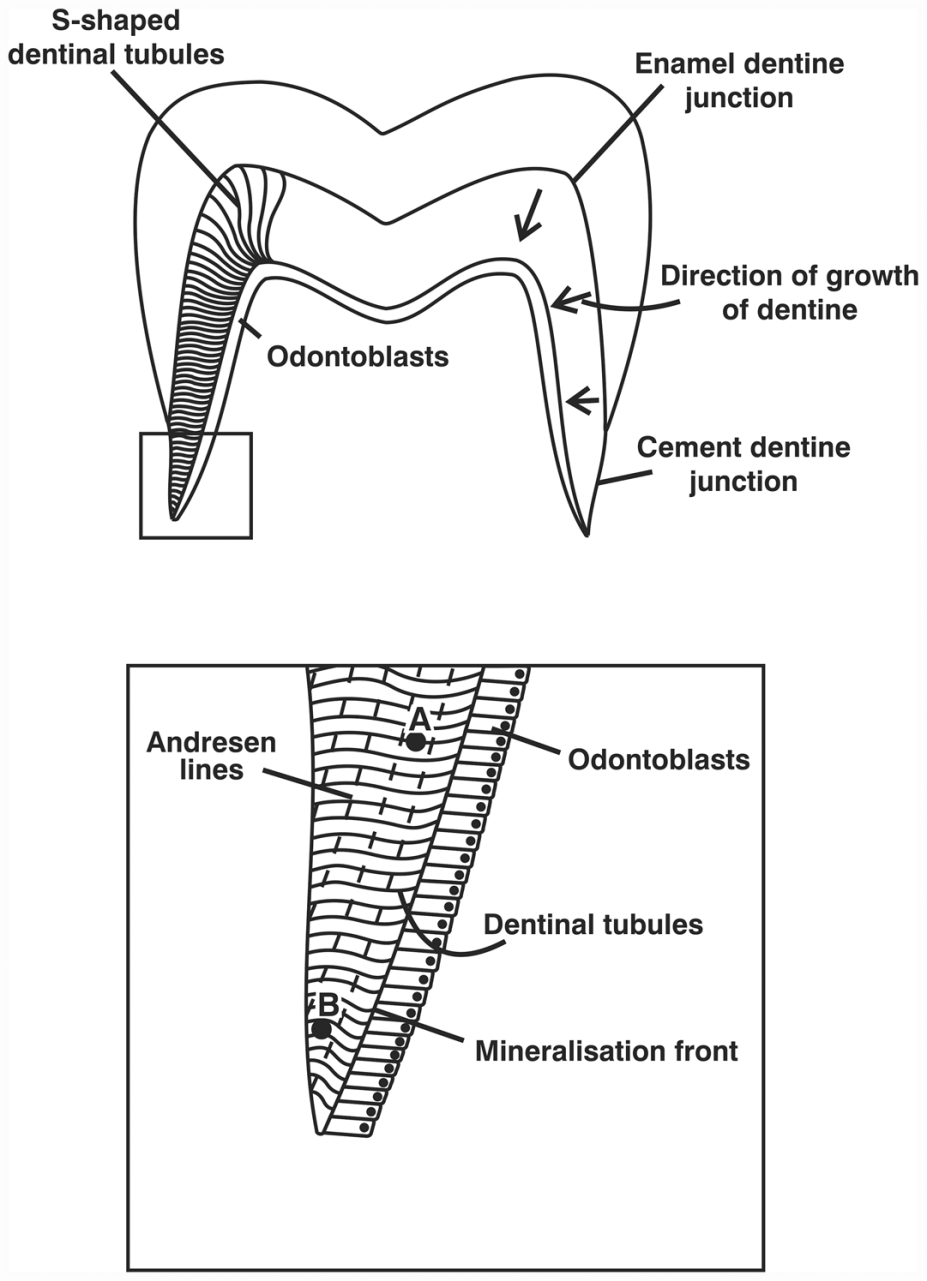
A diagram showing the direction of dentine development in a human molar tooth, the relationship between Andresen bands and the mineralizing front, and points A and B within the same Andresen band [11].

The stable isotope ratios of carbon and nitrogen in bone collagen and bulk dentine collagen have been used extensively over the past thirty years to characterise the childhood and adult diets of archaeological individuals [5,6]. Bone collagen, because of its slow turnover rate, represents a long-term average of the adult diet which can, even in mature adults, date back to adolescence [7]. In contrast, primary dentine is mineralised within 3–4 days of secretion and does not remodel, so represents the diet at the time the tooth was growing [7,8]. Methodological improvements have allowed the analysis of much smaller samples of human dentine, e.g. from a maximum of five per tooth by Fuller et al. [8] to sections of collagen weighing 0.5mg [9], which promise greater temporal resolution. While there are variations in the rate at which dentine is secreted as a permanent human tooth grows [10], the method of measuring 1mm horizontal sections used in this study [11] will produce a rolling average of values. At this resolution, and particularly as the method includes the removal of the circumpulpal dentine to avoid contamination with secondary dentine, there should be no significant effect by assigning the same timing to each section (See Fig 1).

The analysis of incremental dentine has allowed researchers to identify short-term changes in the diet of individuals, and the resulting isotope profiles have been used for the first time for the identification of migrants [11], a detailed analysis of the duration of breastfeeding [12], responses to environmental conditions [13] and potential physiological responses to under-nutrition [14]. The δ^13^C and δ^15^N values of body tissues reflect not only the diet of an individual, but can also be used as evidence for a period of nutritional stress when dietary intake is insufficient for the individual's energy requirements [15,16]. Neuberger et al. [17] report the segmental analysis of hair for forensic investigation of 15 adults and a child believed to have died as the result of food deprivation. They found a trend for the δ^15^N values of the hair segments to rise as body mass index (BMI weight in kilograms divided by the square of height in metres of the individual) reduced. This is consistent with earlier isotopic studies of the δ^15^N values of hair from individuals deprived of food [15,18]. Neuberger et al. [17] also noticed a trend amongst adults for δ^13^C values to be in phase with BMI. This observed drop in δ^13^C was thought to be the result of the breakdown of body fat deposits, which are approximately 3‰ lower than other body tissues such as muscle [19] [17]; in order to maintain the levels of glucose and ketones for energy during a prolonged fast, free fatty acids are obtained from the adipose tissue [20] and may also be used for synthesis of new body tissues in the absence of dietary carbohydrate. This observed fall in δ^13^C in nutritionally-stressed adults complements the findings of Mekota et al. [15] who recorded a rise in δ^13^C and a fall in δ^15^N in the hair of patients recovering from anorexia nervosa once re-feeding started and those of Lehn et al. [21] in the identification of unknown individuals with a poor quality diet. In contrast, Neuberger et al. [17] found the δ^13^C and δ^15^N co-varied in the same direction in hair from a 10 month-old child, suggesting a different metabolic pathway during food deprivation. Cherel et al. [22] discussed the effect of the fasting of King penguins on their δ^13^C blood plasma values, interpreting a rise which they related to lipid content as denoting protein sparing and lipid utilisation during the fasting period. Although energy-partitioning (the relative proportion of energy derived from protein and fat stores) varies between individuals, and is influenced by the pre-starvation percentage of body fat to lean mass, both stores are utilised during prolonged fasting [23].

In the absence of historical documents, identifying famine in a past population presents challenges for the archaeologist. Archaeological contexts may yield evidence for short-term deviations from the usual diet such as inclusion of unusual plants or alterations to butchery practices [24]. Evidence for a catastrophic event in prehistory may hinge upon demography of the cemetery population [25] or archaeological evidence for a single burial event. The osteological analysis of human remains for evidence of malnourishment such as rickets and scurvy and indicators of systemic stress such as enamel hypoplasia, Harris lines and decreased stature all contribute to the health status of individuals [26]. However, Wood et al. [27] pointed out that those who succumb to a rapid death will not survive long enough for the body to produce any bony markers for the causative agent. Those who die from acute diseases associated with famine such as typhus and dysentery will thus appear to have been healthy [28]. Equally, any change in δ^13^C and δ^15^N values resulting from a lack of food and leading shortly to death (which can take as little as 2 months [29]) will not be measurable in bone collagen due to long turnover times and averaging [7]. Collagen turnover in individuals who survived periods of famine will also eventually dilute and obscure any isotopic evidence for under-nutrition. This led researchers to conclude it was not possible to identify famine with isotopic analysis of archaeological bone [30,31]. However, starvation is not the most common cause of death [24]: during periods of nutritional stress the population becomes more susceptible to infections and thus it may be difficult to discriminate between an apparent famine and epidemic disease. Morgan [32] concludes that in the absence of historical evidence, secure dating, and isotopic evidence for dietary physiological changes, the combination of all other available archaeological evidence may still not distinguish between catastrophic death from famine and from epidemic disease, or from a combination of both.

As an historical event, the Great Irish Famine of 1845–52 has been extensively researched and interpreted over the last 150 years. Historians and social commentators have used a wealth of contemporary documents to explore the political, social and environmental factors which combined to turn this particular series of crop failures into a devastating disaster. However, Orser [33] carried out the first archaeological excavation of 19^th^-century Great-Famine-period dwellings in Ireland in rural County Roscommon and made the point that notwithstanding all the contemporary documents we know very little about the rural poor, and there have been few subsequent opportunities to examine the archaeological record from this period in Ireland. The excavation of the Kilkenny Union workhouse famine cemetery by Margaret Gowen & Co. Ltd. from January to June 2006 allowed careful and respectful analysis of the remains of 970 individuals. The minute books for the workhouse indicate the burial ground was in use between August 1847 and March 1851. The workhouse records from this period, and osteological interpretations of the health and disease of the famine cemetery population, have been published by Geber [34,35] and constitute an insightful analysis of the experiences of the Irish poor at this time. This site represents a unique opportunity to examine the effects of an historical famine on the victims both in terms of their observable bony pathology and of stable isotope analysis of their skeletal tissues to investigate diet and physiology recorded in the δ^13^C and δ^15^N values compared with the historical evidence. Such data from people who were nutritionally deprived to such a degree is extremely rare and the opportunity to use their untold stories is of significant import for the assessment of both modern and ancient individuals where nutritional stress may be suspected and soft tissues are not available.

## Materials and Methods

A single permanent tooth was collected from 20 individuals excavated from the Kilkenny Union workhouse famine cemetery, designated numbers KUW 1 to 20. The stable isotope values from incremental dentine collagen were compared with those from the bulk rib bone collagen reported in Beaumont et al. [36]. These individuals were chosen to provide a range of ages and sex, but represent only a small sample of the 970 individuals recorded. Permission was granted for the scientific analysis of these individuals by the Irish Antiquities section of the National Museum of Ireland. All the skeletal remains were re-interred at the Famine Memorial Garden at Goods Shed Square, McDonagh Junction shopping centre, Kilkenny, Ireland, in 2010.

### Methods

In each case incremental dentine collagen was prepared from the full length of a single root or a full longitudinal root section. The tooth notation, sex (if known) and age at death as determined by Geber [36] is recorded for each individual in Table 1 and the isotopic data shown in Table 2. The incremental data for KUW 2, 3 and 8 and KUW 1, 4, 9, 12, 13, 14 and 16 have been previously published [9, 14]. The teeth were cut into 1 mm sections [11] and an approximate age was assigned to each dentine segment [37]. This method allows us to investigate isotopic changes over short periods, approximately 9 months in permanent teeth, although due to the averaging of values (see Fig 1) there will be some attenuation of the profiles.

**Table 1:** Tooth notation, developmental stage of teeth, age at death and sex of individuals [36] from Kilkenny Union workhouse.

Skeleton number	Age	Sex	tooth	stage of tooth development
KUW 1	23–37 years	M	M1	Complete
KUW 2	29–48 years	M	M2	Complete
KUW 3	39–70 years	M	M2	Complete
KUW 4	13 years	0	M2	Rt3/4
KUW 5	21–38 years	F	PM2	Complete
KUW 6	21–35 years	M	M2	Complete
KUW 7	38–63 years	M	M2	Complete
KUW 8	29–51 years	F	M2	Complete
KUW 9	18–28 years	F	M1	Complete
KUW 10	8–9 years	0	M2	Rt1/2
KUW 11	7 years	0	M2	Rt1/4
KUW 12	5–6 years	0	M1	Rt1/2
KUW 13	6 years	0	M1	Rt1/2
KUW 14	7 years	0	M2	Rt1/4
KUW 15	20–30 years	F	M2	Complete
KUW 16	5–6 years	0	M1	Rt1/2
KUW 17	30–55 years	F	PM2	Complete
KUW 18	32–52 years	M	M2	Complete
KUW 19	35–40 years	M	M2	Complete
KUW 20	37–63 years	M	M2	Complete

**Table 2:** Isotope data and collagen quality indicators for dentine sections from teeth from Kilkenny Union Workhouse (KUW 1,4,9,12,13,14,16 previously published in Beaumont et al. 2015[14]).

Sample number	δ^13^C (‰)	δ^15^N (‰)	%C	%N	C:N	Age in years
KUW 1 M1 1	-20.4	11.4	41.9	15.5	3.2	0.5
KUW 1 M1 2	-20.2	11.5	65.8	24.7	3.1	1.2
KUW 1 M1 3	-19.9	11.6	47.1	17.5	3.1	1.9
KUW 1 M1 4	-20.0	11.4	45.6	16.9	3.2	2.5
KUW 1 M1 5	-19.9	11.5	40.4	15.0	3.2	3.2
KUW 1 M1 6	-19.9	11.5	41.7	15.4	3.2	3.9
KUW 1 M1 7	-20.1	11.2	45.4	16.7	3.2	4.6
KUW 1 M1 8	-20.0	11.2	41.0	15.0	3.2	5.3
KUW 1 M1 9	-20.0	11.3	38.4	14.2	3.2	5.9
KUW 1 M1 10	-19.9	11.5	42.2	15.5	3.2	6.6
KUW 1 M1 11	-19.9	11.5	48.8	17.8	3.2	7.3
KUW 1 M1 12	-20.0	11.7	49.4	18.0	3.2	8.0
KUW 1 M1 13	-19.7	12.2	39.5	14.6	3.2	8.6
KUW 1 M1 14	-20.2	11.9	41.2	14.9	3.2	9.3
KUW 2 M2 1	-20.8	11.3	40.8	14.6	3.2	2.5
KUW 2 M2 2	-20.9	10.9	43.7	15.6	3.3	3.3
KUW 2 M2 3	-21.0	10.9	40.6	14.7	3.2	4.1
KUW 2 M2 4	-20.9	11.0	41.4	14.8	3.2	4.9
KUW 2 M2 5	-20.9	11.2	40.4	14.3	3.3	5.7
KUW 2 M2 6	-20.9	11.0	60.8	21.8	3.3	6.6
KUW 2 M2 7	-21.1	10.7	41.3	14.6	3.3	7.4
KUW 2 M2 8	-20.9	11.0	63.7	22.9	3.3	8.2
KUW 2 M2 9	-20.7	11.3	42.3	14.8	3.3	9.0
KUW 2 M2 10	-20.7	11.3	40.7	14.4	3.3	9.8
KUW 2 M2 11	-20.9	11.6	41.3	14.8	3.3	10.6
KUW 2 M2 12	-20.9	11.6	41.3	14.8	3.3	11.4
KUW 2 M2 13	-20.9	11.4	42.3	15.1	3.3	12.2
KUW 2 M2 14	-20.9	11.4	40.0	14.3	3.3	13.0
KUW 2 M2 15	-21.1	11.2	40.6	14.5	3.3	13.8
KUW 2 M2 16	-21.2	10.8	41.0	14.6	3.3	14.7
KUW 2 M2 17	-21.0	10.6	40.5	14.4	3.3	15.5
KUW 3 M2 1	-21.1	10.2	41.8	14.7	3.3	2.5
KUW 3 M2 2	-20.8	10.5	41.0	14.8	3.2	3.3
KUW 3 M2 3	-20.9	10.6	40.7	14.8	3.2	4.1
KUW 3 M2 4	-21.2	11.0	40.8	14.7	3.2	4.9
KUW 3 M2 5	-20.9	10.9	41.2	14.9	3.2	5.7
KUW 3 M2 6	-20.8	10.7	40.9	14.8	3.2	6.6
KUW 3 M2 7	-20.2	10.9	43.9	15.8	3.2	7.4
KUW 3 M2 8	-20.4	11.0	37.5	13.5	3.3	8.2
KUW 3 M2 9	-20.2	11.1	39.4	14.2	3.2	9.0
KUW 3 M2 10	-20.2	11.0	41.4	14.8	3.3	9.8
KUW 3 M2 11	-20.2	11.0	40.9	14.7	3.2	10.6
KUW 3 M2 12	-20.3	11.1	37.1	13.8	3.1	11.4
KUW 3 M2 13	-20.5	11.1	42.1	14.9	3.3	12.2
KUW 3 M2 14	-20.6	11.3	41.0	14.6	3.3	13.0
KUW 3 M2 15	-20.5	11.2	40.7	14.5	3.3	13.8
KUW 3 M2 16	-20.6	11.4	41.1	14.6	3.3	14.7
KUW 3 M2 17	-20.7	11.8	42.4	14.7	3.4	15.5
KUW 4 M2 1	-21.0	10.1	59.9	22.2	3.1	2.5
KUW 4 M2 2	-20.9	10.1	58.3	21.9	3.1	3.2
KUW 4 M2 3	-21.3	9.2	58.2	21.9	3.1	4.0
KUW 4 M2 4	-21.4	9.8	59.2	22.2	3.1	4.7
KUW 4 M2 5	-21.3	10.5	57.4	21.6	3.1	5.4
KUW 4 M2 6	-21.2	10.7	58.0	21.8	3.1	6.2
KUW 4 M2 7	-21.0	10.2	56.2	21.1	3.1	6.9
KUW 4 M2 8	-21.2	10.1	55.2	20.6	3.1	7.6
KUW 4 M2 9	-20.6	9.9	67.3	25.1	3.1	8.3
KUW 4 M2 10	-20.1	9.7	61.0	22.9	3.1	9.1
KUW 4 M2 11	-19.0	9.9	57.9	21.5	3.1	9.8
KUW 4 M2 12	-17.6	10.0	59.9	22.6	3.1	10.5
KUW 4 M2 13	-15.5	9.9	58.2	21.8	3.1	11.3
KUW 4 M2 14	-14.7	10.0	58.7	21.4	3.2	12.0
KUW 5 PM2 1	-20.5	10.7	34.0	12.4	3.2	3.5
KUW 5 PM2 2	-19.9	9.7	41.9	15.6	3.1	4.4
KUW 5 PM2 3	-19.6	9.3	43.2	16.1	3.1	5.2
KUW 5 PM2 4	-19.7	9.4	42.4	15.9	3.1	6.1
KUW 5 PM2 5	-20.2	9.9	42.6	15.9	3.1	6.9
KUW 5 PM2 6	-20.5	10.2	56.4	21.4	3.1	7.8
KUW 5 PM2 7	-20.5	10.4	42.7	16.2	3.1	8.6
KUW 5 PM2 8	-20.5	10.9	42.2	15.9	3.1	9.5
KUW 5 PM2 9	-20.5	10.9	41.2	15.5	3.1	10.3
KUW 5 PM2 10	-20.3	10.8	42.9	16.1	3.1	11.2
KUW 5 PM2 11	-20.1	10.7	40.1	15.1	3.1	12.0
KUW 5 PM2 12	-19.6	10.4	38.2	14.2	3.1	12.9
KUW 5 PM2 13	-19.8	10.0	29.7	10.2	3.4	13.7
KUW 6 M1 1	-20.5	11.5	37.5	13.8	3.2	0.5
KUW 6 M1 2	-20.8	11.7	41.5	15.2	3.2	1.0
KUW 6 M1 3	-21.0	11.8	41.7	15.3	3.2	1.5
KUW 6 M1 4	-21.0	12.0	42.2	15.5	3.2	1.9
KUW 6 M1 5	-20.8	12.3	42.0	15.4	3.2	2.4
KUW 6 M1 6	-20.6	12.3	41.5	15.2	3.2	2.9
KUW 6 M1 7	-20.2	12.1	42.8	15.8	3.2	3.4
KUW 6 M1 8	-20.1	12.0	42.0	15.4	3.2	3.9
KUW 6 M1 9	-20.1	12.1	42.3	15.6	3.2	4.3
KUW 6 M1 10	-20.2	11.9	42.0	15.4	3.2	4.8
KUW 6 M1 11	-20.3	11.8	42.1	15.5	3.2	5.3
KUW 6 M1 12	-20.5	11.6	42.4	15.6	3.2	5.8
KUW 6 M1 13	-20.7	11.8	42.3	15.6	3.2	6.3
KUW 6 M1 14	-20.5	11.7	41.9	15.4	3.2	6.7
KUW 6 M1 15	-20.2	11.7	41.2	15.2	3.2	7.2
KUW 6 M1 16	-20.3	11.7	40.5	14.9	3.2	7.7
KUW 6 M1 17	-20.4	12.0	50.2	18.6	3.2	8.2
KUW 6 M1 18	-20.5	12.2	40.4	14.8	3.2	8.7
KUW 6 M1 19	-20.6	12.2	36.7	13.4	3.2	9.1
KUW 7 M2 1	-21.7	11.4	54.9	20.2	3.2	2.5
KUW 7 M2 2	-21.4	10.4	55.1	20.5	3.1	3.4
KUW 7 M2 3	-21.2	10.4	76.6	28.8	3.1	4.2
KUW 7 M2 4	-21.0	10.7	51.0	19.1	3.1	5.1
KUW 7 M2 5	-20.8	10.6	54.7	20.6	3.1	6.0
KUW 7 M2 6	-20.8	10.5	55.2	20.7	3.1	6.8
KUW 7 M2 7	-20.8	10.5	74.2	27.8	3.1	7.7
KUW 7 M2 8	-20.7	10.4	55.6	20.9	3.1	8.6
KUW 7 M2 9	-20.7	10.7	58.0	21.9	3.1	9.4
KUW 7 M2 10	-20.7	10.6	56.0	21.1	3.1	10.3
KUW 7 M2 11	-20.8	10.6	54.9	20.7	3.1	11.2
KUW M2 7 12	-20.9	10.7	42.0	15.0	3.3	12.0
KUW M2 7 13	-20.7	10.6	37.1	13.4	3.2	12.9
KUW M2 7 14	-20.7	10.5	46.6	16.7	3.3	13.8
KUW M2 7 15	-20.8	11.0	41.0	14.8	3.2	14.6
KUW M2 7 16	-20.9	11.2	53.6	20.7	3.2	15.5
KUW 8 M2 1	-20.8	11.9	42.7	15.3	3.3	2.5
KUW 8 M2 2	-20.4	11.5	42.1	15.4	3.2	3.3
KUW 8 M2 3	-20.4	11.2	42.3	15.5	3.2	4.1
KUW 8 M2 4	-20.5	10.8	42.3	15.5	3.2	4.9
KUW 8 M2 5	-20.3	10.7	38.9	14.2	3.2	5.7
KUW 8 M2 6	-19.7	10.4	41.5	15.1	3.2	6.6
KUW 8 M2 7	-19.8	10.5	42.9	15.7	3.2	7.4
KUW 8 M2 8	-20.0	10.7	41.0	14.9	3.2	8.2
KUW 8 M2 9	-20.1	11.1	40.5	14.8	3.2	9.0
KUW 8 M2 10	-19.8	10.9	41.7	15.2	3.2	9.8
KUW 8 M2 11	-19.8	10.9	40.4	14.7	3.2	10.6
KUW 8 M2 12	-20.1	10.6	40.0	14.5	3.2	11.4
KUW 8 M2 13	-20.4	10.6	43.0	15.7	3.2	12.2
KUW 8 M2 14	-20.5	10.6	43.5	15.9	3.2	13.0
KUW 8 M2 15	-20.6	11.0	42.5	15.4	3.2	13.8
KUW 8 M2 16	-20.6	11.3	42.6	15.4	3.2	14.7
KUW 8 M2 17	-20.7	11.2	42.8	15.5	3.2	15.5
KUW 9 M1 1	-20.4	10.2	11.9	5.0	2.8	0.5
KUW 9 M1 2	-20.7	9.5	7.9	3.4	2.7	1.2
KUW 9 M1 3	-20.6	10.0	13.6	5.6	2.8	1.9
KUW 9 M1 4	-20.5	10.5	16.5	6.6	2.9	2.5
KUW 9 M1 5	-20.4	11.0	13.6	5.7	2.8	3.2
KUW 9 M1 6	-20.5	11.4	25.6	10.1	3.0	3.9
KUW 9 M1 7	-20.6	11.5	9.5	3.7	2.9	4.6
KUW 9 M1 8	-20.7	11.6	16.7	6.7	2.9	5.3
KUW 9 M1 9	-20.7	11.5	17.3	7.0	2.8	5.9
KUW 9 M1 10	-20.8	11.3	6.6	2.6	3.0	6.6
KUW 9 M1 11	-20.8	11.0	20.5	8.0	3.0	7.3
KUW 9 M1 12	-20.8	11.1	26.8	10.5	3.0	8.0
KUW 9 M1 13	-20.7	11.3	20.7	8.1	3.0	8.6
KUW 9 M1 14	-20.8	11.4	11.2	4.5	2.9	9.3
KUW 10 M2 1	-21.1	10.5	41.3	15.2	3.2	2.5
KUW 10 M2 2	-20.9	10.8	41.0	14.9	3.2	3.2
KUW 10 M2 3	-20.3	10.4	42.2	15.5	3.2	3.9
KUW 10 M2 4	-20.6	10.5	41.6	15.2	3.2	4.7
KUW 10 M2 5	-20.7	10.5	41.2	15.2	3.2	5.4
KUW 10 M2 6	-20.8	10.5	40.6	15.0	3.2	6.1
KUW 10 M2 7	-20.8	10.5	41.4	15.2	3.2	6.8
KUW 10 M2 8	-20.7	10.8	40.7	15.0	3.2	7.5
KUW 10 M2 9	-20.9	11.3	40.5	14.6	3.2	8.3
KUW 10 M2 10	-20.5	11.7	41.7	15.3	3.2	9.0
KUW 11 M2 1	-20.5	11.5	44.5	15.9	3.3	2.5
KUW 11 M2 2	-20.6	10.9	39.9	14.5	3.2	3.3
KUW 11 M2 3	-20.2	10.7	43.5	15.8	3.2	4.0
KUW 11 M2 4	-18.9	10.9	41.0	15.1	3.2	4.8
KUW 11 M2 5	-17.4	11.0	41.6	14.9	3.2	5.5
KUW 12 M1 1	-19.7	11.1	33.1	12.3	3.5	0.5
KUW 12 M1 2	-18.8	10.4	50.4	19.0	3.2	1.3
KUW 12 M1 3	-18.6	10.3	38.3	14.2	3.2	2.1
KUW 12 M1 4	-19.1	10.4	47.0	17.5	3.1	2.9
KUW 12 M1 5	-19.3	10.7	36.5	13.4	3.0	3.7
KUW 12 M1 6	-19.4	10.7	36.5	13.6	2.9	4.5
KUW 12 M1 7	-19.6	11.2	43.3	15.2	3.0	5.2
KUW 12 M1 8	-19.7	11.1	43.7	15.2	3.4	6.0
KUW 13 M1 1	-19.9	14.3	35.3	12.8	3.2	0.5
KUW 13 M1 2	-19.7	13.0	49.8	18.4	3.2	1.4
KUW 13 M1 3	-18.8	11.8	34.3	12.6	3.2	2.3
KUW 13 M1 4	-17.6	10.7	27.9	10.3	3.2	3.3
KUW 13 M1 5	-16.5	10.4	28.3	10.4	3.2	4.2
KUW 13 M1 6	-16.4	9.8	37.8	13.7	3.2	5.1
KUW 13 M1 7	-18.3	12.3	31.8	11.5	3.2	6.0
KUW 14 M2 1	-20.6	11.3	40.7	15.0	3.2	2.5
KUW 14 M2 2	-20.0	11.2	41.1	15.2	3.2	3.7
KUW 14 M2 3	-19.7	10.9	41.5	15.3	3.2	4.8
KUW 14 M2 4	-20.0	11.2	41.4	15.3	3.2	6.0
KUW 14 M2 5	-19.2	11.0	41.4	15.2	3.2	7.2
KUW 14 M2 6	-18.1	11.1	41.9	15.3	3.2	8.4
KUW 14 M2 7	-17.0	11.1	41.7	15.1	3.2	9.5
KUW 15 M2 1	-20.5	12.1	41.2	15.0	3.2	2.5
KUW 15 M2 2	-20.3	10.9	42.9	15.6	3.2	3.3
KUW 15 M2 3	-20.1	10.6	41.8	15.4	3.2	4.1
KUW 15 M2 4	-19.9	10.6	44.5	16.3	3.2	4.9
KUW 15 M2 5	-19.8	10.6	43.3	15.9	3.2	5.7
KUW 15 M2 6	-19.9	10.5	42.7	15.7	3.2	6.6
KUW 15 M2 7	-20.1	10.5	43.4	16.0	3.2	7.4
KUW 15 M2 8	-19.9	10.4	43.1	15.7	3.2	8.2
KUW 15 M2 9	-19.9	10.5	40.7	14.9	3.2	9.0
KUW 15 M2 10	-19.8	10.5	47.2	17.3	3.2	9.8
KUW 15 M2 11	-19.8	10.5	36.3	13.2	3.2	10.6
KUW 15 M2 12	-20.0	10.5	50.9	18.7	3.2	11.4
KUW 15 M2 13	-20.3	10.5	42.5	15.5	3.2	12.2
KUW 15 M2 14	-20.4	10.6	43.5	15.9	3.2	13.0
KUW 15 M2 15	-20.3	11.1	43.4	15.9	3.2	13.8
KUW 15 M2 16	-20.0	11.3	43.4	15.9	3.2	14.7
KUW 15 M2 17	-19.5	11.6	44.4	16.4	3.2	15.5
KUW 16 M1 1	-19.5	11.8	50.1	18.3	3.2	0.5
KUW 16 M1 2	-19.3	11.6	52.5	19.2	3.2	1.3
KUW 16 M1 3	-20.6	10.7	50.3	18.5	3.2	2.0
KUW 16 M1 4	-21.0	10.6	55.1	19.5	3.3	2.8
KUW 16 M1 5	-20.3	10.6	56.4	20.9	3.2	3.5
KUW 16 M1 6	-19.8	10.6	55.0	20.4	3.1	4.3
KUW 16 M1 7	-19.6	10.8	57.4	20.0	3.3	5.0
KUW 16 M1 8	-18.3	11.1	31.3	11.7	3.1	5.8
KUW 17 PM2 1	-21.1	12.7	44.7	16.7	3.1	3.5
KUW 17 PM2 2	-20.5	12.2	44.2	16.6	3.1	4.1
KUW 17 PM2 3	-20.9	11.6	45.2	17.0	3.1	4.7
KUW 17 PM2 4	-21.4	11.1	45.6	17.3	3.1	5.2
KUW 17 PM2 5	-20.9	11.4	44.4	16.7	3.1	5.8
KUW 17 PM2 6	-20.9	12.3	129.2	49.1	3.1	6.4
KUW 17 PM2 7	-21.0	12.3	54.2	19.8	3.2	7.0
KUW 17 PM2 8	-21.0	12.2	54.5	20.1	3.2	7.6
KUW 17 PM2 9	-20.9	11.8	54.1	19.9	3.2	8.1
KUW 17 PM2 10	-20.9	11.3	54.1	20.0	3.2	8.7
KUW 17 PM2 11	-20.9	11.1	51.9	19.1	3.2	9.3
KUW 17 PM2 12	-20.8	11.1	55.1	20.3	3.2	9.9
KUW 17 PM2 13	-20.6	11.4	55.2	20.4	3.1	10.5
KUW 17 PM2 14	-20.6	11.6	56.4	21.0	3.1	11.0
KUW 17 PM2 15	-20.7	11.6	55.4	20.6	3.1	11.6
KUW 17 PM2 16	-20.6	11.3	54.1	20.1	3.1	12.2
KUW 17 PM2 17	-20.6	11.5	54.6	20.2	3.2	12.8
KUW 17 PM2 18	-20.6	11.6	54.6	20.2	3.2	13.4
KUW 17 PM2 19	-20.7	11.7	55.0	20.3	3.2	13.9
KUW 17 PM2 20	-20.9	11.8	53.5	19.5	3.2	14.5
KUW 18 M2 1	-20.0	11.9	45.4	16.8	3.1	2.5
KUW 18 M2 2	-20.1	11.9	44.0	16.3	3.1	3.7
KUW 18 M2 3	-20.1	11.8	43.5	16.3	3.1	4.9
KUW 18 M2 4	-20.2	12.0	43.7	16.2	3.2	6.0
KUW 18 M2 5	-20.1	12.0	45.3	16.9	3.1	7.2
KUW 18 M2 6	-20.3	11.9	44.3	16.4	3.2	8.4
KUW 18 M2 7	-20.3	12.2	44.0	16.3	3.2	9.6
KUW 18 M2 8	-20.3	12.2	44.2	16.4	3.2	10.8
KUW 18 M2 9	-20.4	12.4	42.4	15.6	3.2	11.9
KUW 18 M2 10	-20.4	12.4	40.8	15.2	3.2	13.1
KUW 18 M2 11	-20.6	11.4	48.1	19.0	3.0	14.3
KUW 18 M2 12	-20.2	11.9	43.5	16.1	3.1	15.5
KUW 19 M2 2	-21.1	11.1	46.3	17.2	3.1	3.3
KUW 19 M2 3	-20.9	11.2	46.2	17.3	3.1	4.1
KUW 19 M2 4	-20.6	11.2	44.5	16.7	3.1	4.9
KUW 19 M2 5	-20.6	11.0	45.3	17.0	3.1	5.7
KUW 19 M2 6	-20.7	10.8	44.8	16.9	3.1	6.6
KUW 19 M2 7	-20.8	10.5	44.7	16.7	3.1	7.4
KUW 19 M2 8	-20.6	10.6	44.4	16.6	3.1	8.2
KUW 19 M2 9	-20.6	10.7	44.8	16.6	3.1	9.0
KUW 19 M2 10	-20.6	10.5	44.5	16.7	3.1	9.8
KUW 19 M2 11	-20.5	10.5	46.2	17.3	3.1	10.6
KUW 19 M2 12	-20.5	10.5	44.5	16.6	3.1	11.4
KUW 19 M2 13	-20.5	10.7	46.4	17.4	3.1	12.2
KUW 19 M2 14	-20.7	10.6	44.5	16.6	3.1	13.0
KUW 19 M2 15	-20.7	11.0	42.1	15.7	3.1	13.8
KUW 19 M2 16	-20.3	12.0	43.8	16.4	3.1	14.7
KUW 19 M2 17	-20.0	12.7	43.9	16.2	3.2	15.5
KUW 20 M2 1	-20.5	11.6	43.1	15.5	3.2	2.5
KUW 20 M2 2	-20.8	11.6	39.1	14.2	3.2	3.4
KUW 20 M2 3	-20.8	11.6	41.8	15.8	3.1	4.2
KUW 20 M2 4	-20.6	11.1	43.3	15.9	3.2	5.1
KUW 20 M2 5	-20.5	11.2	41.9	15.3	3.2	6.0
KUW 20 M2 6	-20.3	11.2	44.8	16.6	3.1	6.9
KUW 20 M2 7	-19.9	11.4	40.6	14.8	3.2	7.7
KUW 20 M2 8	-19.8	11.5	39.0	14.2	3.2	8.6
KUW 20 M2 9	-20.1	11.5	43.8	16.0	3.2	9.5
KUW 20 M2 10	-20.7	11.7	42.1	15.3	3.2	10.3
KUW 20 M2 11	-20.9	11.7	39.2	14.3	3.2	11.2
KUW 20 M2 12	-20.8	11.4	41.4	15.2	3.2	12.1
KUW 20 M2 13	-20.7	11.3	42.5	15.5	3.2	12.9
KUW 20 M2 14	-20.5	11.2	43.4	15.8	3.2	13.8
KUW 20 M2 15	-20.5	11.4	42.5	15.4	3.2	14.7
KUW 20 M2 16	-20.4	11.7	32.9	11.9	3.2	15.6

Collagen was prepared using the modified Longin method [38]. Surface debris was removed by air-abrasion and samples were demineralised in 0.5M hydrochloric acid at 4°C. Each root was then divided into 1mm sections using a scalpel. Each section was placed in sealed microtubes, heated in pH3 acidified water at 70°C for 48 hours, allowing the collagen fibrils to denature. Filtration of dentine has been shown to be unnecessary due to the lack of exogenous debris (compared to bone) and the very high collagen yields [9,11]. Samples were centrifuged prior to freezing and freeze-drying. The dentine samples were combusted in a Thermo Flash EA 1112 and the separated N_2_ and CO_2_ was introduced to a Delta plus XL via a Conflo III interface. This instrument was used for dentine samples because it can analyse small (0.5 mg) samples.

Samples were measured in duplicate in the University of Bradford Stable Light Isotope Laboratory and compared with laboratory and international standards that were interspersed throughout each analytical run (Table 3). The international standards were: IAEA 600, CH6, CH7, N1 and N2. The laboratory standards, fish gelatin and bovine liver, were calibrated against the international standards. The C:N ratios obtained from each dentine collagen sample are within the range of 3.1–3.5 recommended by van Klinken [39]. This indicates well-preserved collagen that has not undergone diagenetic alteration. The yields from dentine were between 10–19% by weight before demineralization. The results for both dentine and bone collagen are expressed using the delta (δ) notation in parts per thousand (per mil or ‰) relative to the international standards Vienna-PDB for δ^13^C and AIR for δ^15^N. The analytical error was determined at ±0.2‰ (1sd) or better.

**Table 3:** Carbon and nitrogen isotope ratio values for international and laboratory standards used in this study.

	δ^13^C‰	δ^15^N‰
**International standard**		
IAEA 600	-27.77±0.04	+1.0±0.2
IAEA CH6	-10.45±0.03	
IAEA CH7	-32.15±0.05	
N1		+0.43±0.2
N2		+20.41±0.2
**University of Bradford Stable Light Isotope Laboratory standards**		
Fish gelatine	-15.52	+14.45
BLS	-21.59±0.25	+7.65±0.25

## Results and Discussion

**Fig 2:**
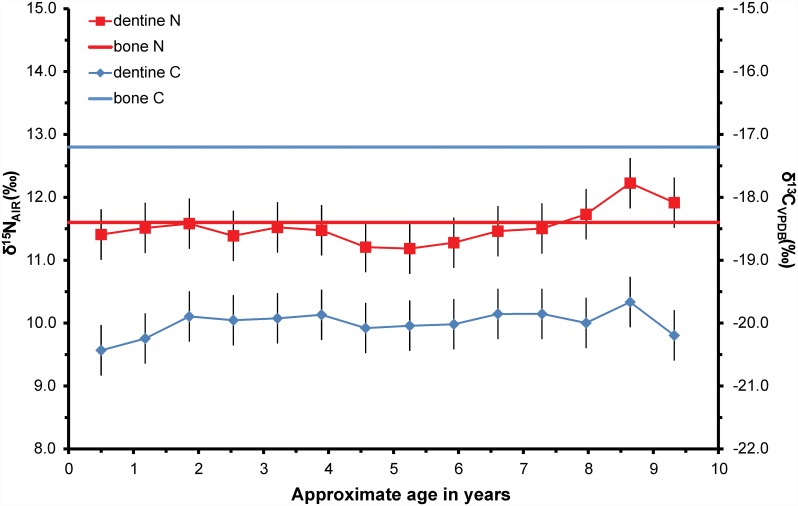
Incremental dentine carbon (δ^13^C) and nitrogen (δ^15^N) isotope ratio profile for KUW 1 an adult who died at ~ 23–37 years of age, i.e. at least 13 years after the tooth finished mineralising. Analytical uncertainty is shown at +/- 0.4‰ (2sd).

The dentine isotope ratios from the adults reflect their childhood diet which is known from historical sources to have been extremely restricted and dominated by potatoes supplemented with a small amount of animal protein. The dentine δ^13^C values for the adult individuals range between -21.7‰ and -19.5‰ (x = -20.5 +/- 0.3‰ 1sd, n = 13); the δ^15^N values range between 9.3‰ and 12.7‰ (x = 11.2 +/- 0.5‰ 1sd, n = 13). The intra-individual range of isotope ratios is smaller: δ^13^C ≤ 1.6‰; δ^15^N ≤ 2.2‰. Although small, these ranges exceed analytical uncertainty (i.e. +/-0.4‰ 2sd) and the δ^13^C and δ^15^N values co-vary as would be expected during minor changes in dietary protein consumption during childhood and adolescence. For example, KUW 1 exhibits low variation (δ^13^C = 0.8‰; δ^15^N = 1‰) in dentine isotope ratio profiles between the ages of 0.5 and 9 years but an incremental δ^15^N rise of 1‰ (which exceeds analytical uncertainty at 2sd), between the ages of 5 and 9 years (Fig 2). The progressive nature of the rise suggests this is a real if small change and not merely random analytical error. Whilst the δ^15^N dentine (childhood) profile is analytically indistinguishable from the rib bone (adult) value, this is not the case for δ^13^C which shows a ~3‰ increase from c. -20‰ in childhood to -17.2‰ in later life. The rise in δ^13^C is not accompanied by a rise in δ^15^N and thus is most likely to be recording a change from a predominantly C_3_ potato-based childhood diet to a diet including C_4_ maize at some time between the age of 9 years and death more than 13 years later. Additional individual plots for adults from Kilkenny Union workhouse which show that their childhood δ^13^C values reflecting a C_3_ potato-based predominantly range from -21‰ and -20‰ are presented in S1, S2 and S3 Figs.

**Fig 3:**
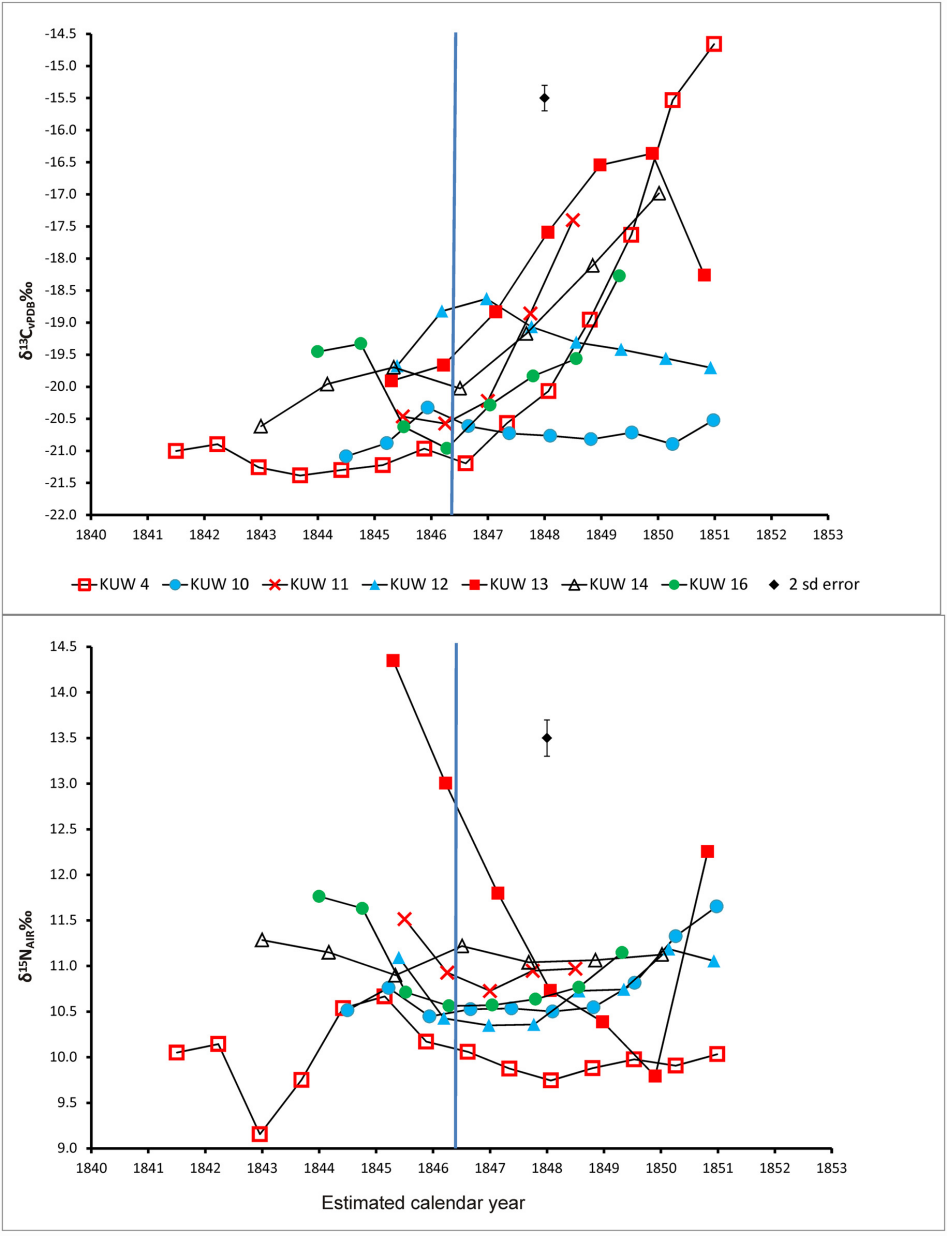
Incremental dentine carbon (δ^13^C) and nitrogen (δ^15^N) isotope ratio profiles for juveniles from Kilkenny Union Workhouse aligned with the estimated calendar year of life: the blue vertical line denotes the introduction of maize as a C_4_ relief food in March 1846.

In contrast, the dentine isotope profiles of the seven children who died whilst their teeth were forming, and which thus track diet up to death with increased temporal resolution, display much larger variations in δ^13^C and δ^15^N (Fig 3). The δ^13^C values for the children range between -21.4‰ and -14.7‰ (x = -19.5 +/- 0.8‰ 1sd, n = 7); the δ^15^N values range between 9.2‰ and 14.3‰ (x = 10.9 +/- 0.5‰ 1sd, n = 7). The intra-individual range of isotope ratios is the same for δ^13^C at ≤ 6.7‰ and marginally smaller for δ^15^N at ≤ 4.6‰. The large shifts in the children’s δ^13^C isotope profiles demonstrate that, with the exception of KUW 10 (a nine year old child), they changed their main calorie intake from C_3_ potatoes to C_4_ maize at some point prior to death. For KUW 4, 11, 13, 14, 16 the δ^13^C profiles were rising immediately prior to death. This is consistent with the documentary evidence relating to the maize-based diet provided in workhouses during the Great Famine [3]. The combined δ^13^C and δ^15^N profiles of these children show that, as expected, the maize-based diet increases the δ^13^C values with *opposing covariance* of the δ^15^N values (i.e. the fall in δ^15^N occurs at the same time as the rise in δ^13^C), reflecting the change to the C_4_ plant-based diet (i.e. the maize pattern) and perhaps suggesting a maize-based diet results in lower δ^15^N values. In Fig 3 the profiles from the children are aligned with two known historical events: the introduction of maize imported from North America in March 1846 and the closure of the cemetery in March 1851. Where possible, the profiles are matched to the introduction of maize by aligning the first rise in δ^13^C with March 1846. In two cases (KUW 10 and KUW 12) this meant that the profile continued to death beyond the closure of the cemetery, which was not possible. When those profiles are aligned with death at 1851, the rise in δ^13^C appears to predict the dietary change, showing that there is some error in the calculation of the age assignment of the dentine sections, or there is averaging due to the overlapping developmental structures (see Fig 1). However, in all cases there is a rise in δ^15^N just prior to the dietary change to maize (Fig 3). Moreover, the δ^15^N values in most children rise just prior to death. The rises in δ^15^N are not due to a higher trophic level diet, as δ^13^C should also rise (although to a much smaller extent); in all cases δ^13^C values remain stable or fall, showing the opposite pattern of *opposing co-variance* (i.e. the famine pattern). Dietary explanations for a rise in δ^15^N values without a corresponding rise in δ^13^C values such as the consumption of freshwater fish or meat from animals raised in a forested environment should be considered but these have not been historically documented for this population. Thus, this pattern appears to be evidence for a period of nutritional stress prior to the introduction of maize as a famine relief food. This is consistent with the findings of Neuberger et al., Lehn et al. and Cherel et al. [17,21,22] that the recycling of body fat stores causes a fall in δ^13^C values in body tissues. Huelseman et al. [40], during their controlled feeding experiment where terrestrial foods were replaced with marine, found that the rise in δ^15^N values in hair keratin was recorded more rapidly than for δ^13^C. Furthermore, the increase in δ^15^N was greater in the individuals with a higher rate of physical activity suggesting that the rise as a result of dietary change was accompanied by a rise associated with nutritional stress.

**Fig 4:**
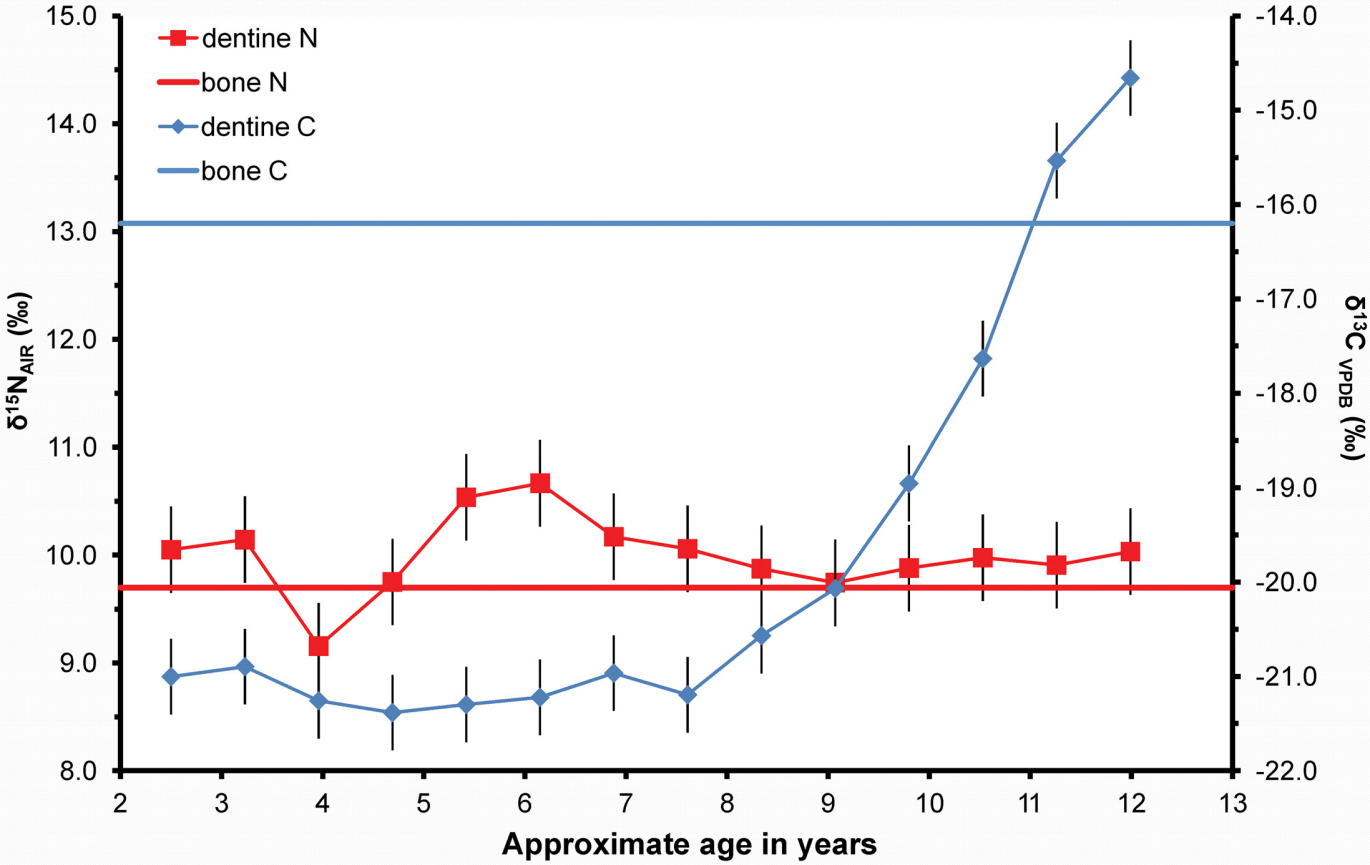
Incremental dentine carbon (δ^13^C) and nitrogen (δ^15^N) isotope ratio profile of KUW 4 a juvenile who died ~13 years of age when the tooth was still mineralising. It shows the opposing covariance famine pattern age 4.5 to 7 years followed by the maize pattern 8.5 to 13 years. Analytical uncertainty is shown at +/- 0.4‰ (2sd).

Both patterns of opposing co-variance are visible in the profile of the juvenile KUW 4 (Fig 4) commencing with low δ^13^C: high δ^15^N of the famine pattern between the ages of 4 and 8 years of age and switching to high δ^13^C: low δ^15^N of the maize pattern between 8 years of age and death. As with the adult KUW 1 (Fig 2), the δ^15^N value for the rib bone correlates well with the dentine values (bone δ^15^N = 9.7‰, mean dentine δ^15^N = 10‰), and they are indistinguishable in the last c. 5 years of life. The δ^15^N value peaks at 10.7‰ at c. 6 years of age which is only 1‰ different from the rib value but is preceded by an incremental rise and followed by an incremental fall suggesting again that this is a subtle but analytically significant change. The shift in δ^13^C between dentine and rib is large: the dentine has a mean value of -21‰ (n = 10) between the ages of 2 and 9 years but the δ^13^C rib value of -16.2‰ clearly reflects the shift to a C_4_ maize diet between the ages of 8 and 9 years. Whilst the rib value falls short of the peri-mortem dentine peak of -14.7‰, showing it was still in the process of equilibrating with the C_4_ diet at death, it sits between the means of the last four increments and the last three increments, i.e. -16.7‰ and -15.9‰ respectively, suggesting the rib value of this c. 13 year old child averages the last c. 3 years of dietary inputs. Additional individual profiles for juveniles from Kilkenny Union workhouse presented in S3 Fig show that, where there has been a C_3_ to C_4_ dietary change prior to death, rib and final dentine increments can record different δ^13^C values where, as in the case of KUW 4, the rib appears to be lagging behind the dentine (e.g. KUW 11,13,16) or, somewhat inexplicably, predicting future dentine values (e.g. KUW 14).

**Fig 5:**
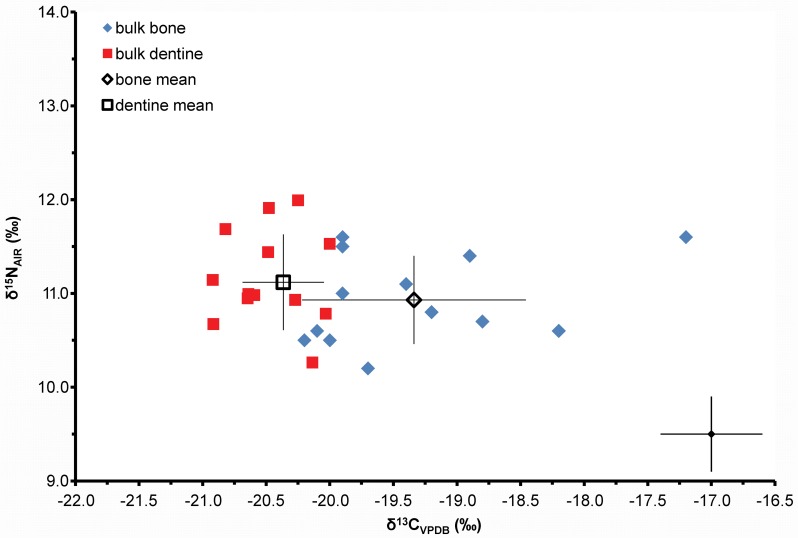
Plot showing the carbon (δ^13^C) and nitrogen (δ^15^N) isotope ratios for the bulk rib bone collagen [36] and whole tooth mean dentine collagen for the adult individuals from Kilkenny Union workhouse. Analytical uncertainty is shown at +/- 0.4‰ (2sd).

The difference between the δ^13^C value of individual adult rib and mean dentine (Δ^13^C) varies from 0.2‰ to 2.8‰ but is, in all cases, positive, i.e. later life values are higher than childhood values (Fig 5) although there is no significant correlation with age at death and Δ^13^C (Pearson r = 0.43, p-value = 0.15). The δ^13^C mean of the ribs is significantly greater than the mean of the bulk dentine (paired t-test p < 0.05, n = 13) and the data provide strong evidence that short-term consumption of C_4_ maize has been variably recorded in the rib collagen through bone turnover during the last few years of life whilst the dentine retains the C_3_ potato-based isotope values of the childhood diet (Fig 5 and S3 Fig). The retention of early life C_3_ isotope values in the adult dentine is supported by the fact that virtually none of the adult dentine δ^13^C values rise above -20‰, the dentine values for adults are significantly less variable than those of the children (adult dentine mean -20.4‰ ± 0.4‰ 1sd; children dentine mean 19.6‰ ± 1.5‰ 1sd, p < 0.05) and that the children's dentine mean is significantly greater than the adult dentine mean (t-test p < 0.05).

As indicated above, the consumption of maize by the adults in the Kilkenny Union workhouse is recorded variably in the ribs and whilst there is no statistically significant correlation with age at death in this relatively small group, it is difficult to conclude what other factors such as onset of maize consumption, activity, nutrition and health may have impacted on the visibility of maize consumption in the adult bones. Equally, the transition from potatoes to maize visible within the dentine δ^13^C values of the children also appears to take several years (e.g. Fig 4) because each sample of dentine, although of far higher temporal resolution than bone, still does not represent a discrete time period but rather a running average due to current sample size requirements and the pattern of appositional dentine growth (Fig 1) [11]. As a result, a step-change in diet from C_3_ to C_4_ will manifest as a rising profile starting with the increment that includes the first C_4_ influenced dentine in the innermost layer. This dentine will also be forming simultaneously in outer dentine layers nearer the tooth apex. If these layers continue to build up with only C_4_-influenced dentine the profile further down the tooth, if not arrested by death, will plateau at the new higher δ^13^C value. However appositional growth means that the amplitude of a *temporary* dietary change *within* a profile will be attenuated and the peak flattened as is suggested for the δ^15^N peak of KUW 4 that occurs between the ages of 5 and 7 years (Fig 4). Here, it is highly likely that the visible peak does not represent the highest short-term δ^15^N dentine value because after this dentine layer was laid down, additional layers would have been added with lower δ^15^N values reducing the averaged value. The true height of the peak is therefore no longer visible at the current sampling resolution as KUW 4 survived this period of stress. This strongly implies that a period of famine may manifest as what might be reasonably considered a fairly minor (i.e. ~1‰) rise in δ^15^N. A further interpretive complication is that increments at the beginning and the end of a tooth profile may record more extreme values because they are subject to only one-way averaging, i.e. dentine formed either solely after or before the increment in question not both, and thus they will have a higher temporal resolution. In the case of the final increments of incomplete tooth roots, such as that of KUW 4 and the other juveniles in this study, the temporal resolution will be even greater because the dentine has not yet reached its final thickness. For most incomplete teeth, the final increment is extremely thin consisting of outer dentine layers only, and thus its isotope ratios will not be ameliorated by dentine that would have been laid down in the future if the individual had survived and the tooth continued to completion. Turning this around, it also implies that the onset of a dietary change from C_3_ to C_4_ will be heralded at an earlier age in the profiles presented here than it actually occurred because the inner, later forming layers of dentine will cause the profiles to rise. These observations suggest that similar dietary or physiological changes may manifest differently in dentine isotope profiles depending on whether the change occurred within a completed tooth root and was survived, or at the end of a profile in a tooth where development was arrested at death. Temporary changes in δ^13^C as a result of a change in diet or increases in δ^15^N as a result of famine may, therefore, be far more subtle and of suppressed amplitude than might be anticipated or indeed obtained from the incomplete tooth roots of children. Thinning the dentine of fully formed teeth from the pulp cavity prior to sectioning should reduce the impact of later appositional deposition of dentine and thus increase the temporal resolution of the isotope profiles.

**Fig 6:**
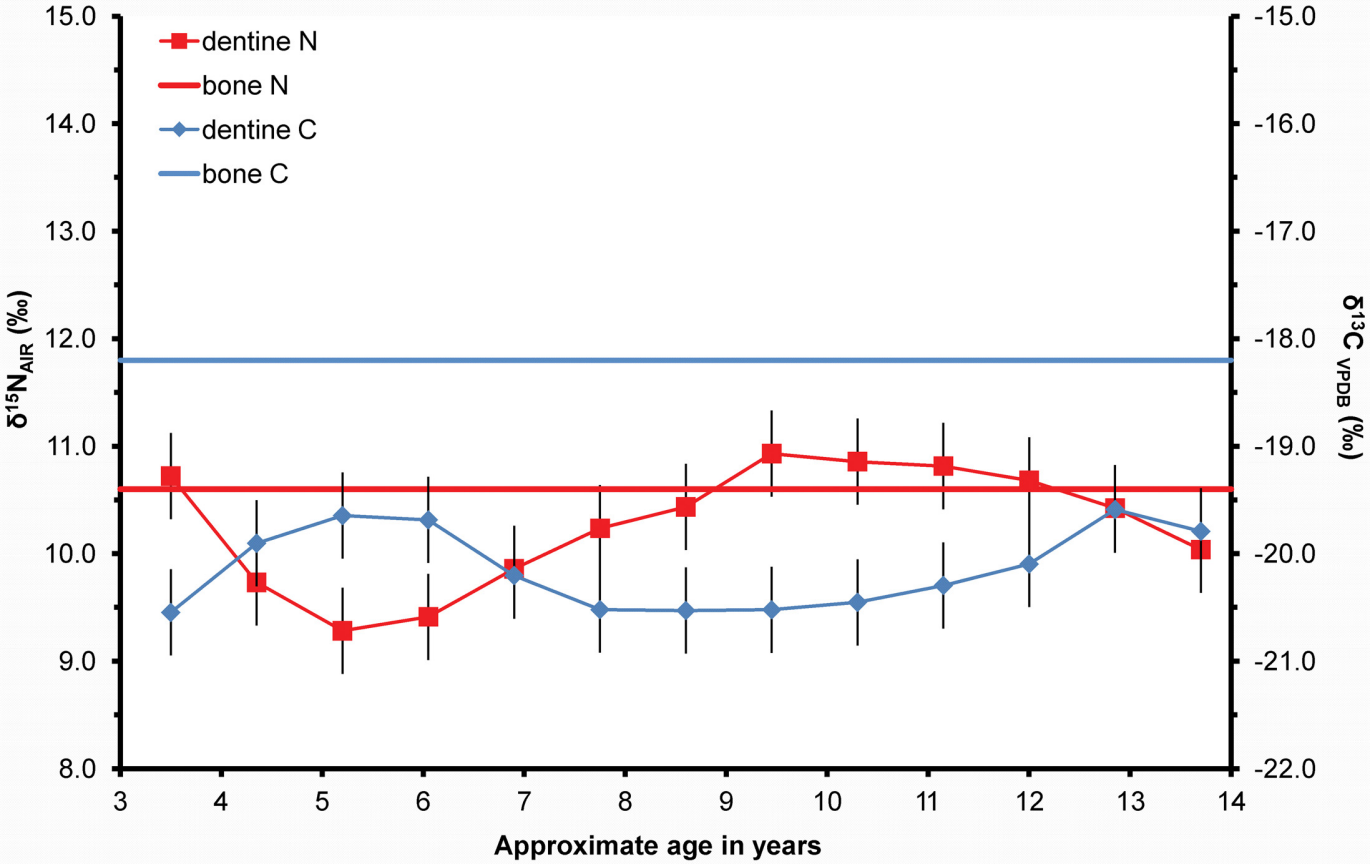
Incremental dentine carbon (δ^13^C) and nitrogen (δ^15^N) isotope ratio profile of KUW 5 an adult female who died at ~21 to 38 years of age, i.e. at least 7 years after the tooth finished mineralising. It shows the opposing covariance maize pattern age 4 to 7 years, and the famine pattern age 8.5 to 12 years. Analytical uncertainty is shown at +/- 0.4‰ (2sd).

The Great Irish Famine of 1845–52 was not the first major famine in Ireland nor was the use of maize as a relief food in March 1846 the first major importation of maize to Ireland. Crawford [41] demonstrates that maize was imported in quantity in 1801 and again in 1827 to supply cheap food to the Irish poor. During 1827, when there were widespread crop failures, Humphrey O’Sullivan, a schoolteacher in Callan, County Kilkenny, records in his diary that 'Indian meal' imported from America was being distributed in an attempt to “*keep down the cost of living for the poor*” [41]. Whilst it is only possible to speculate about the actual cause of death for the people buried in the Kilkenny Union workhouse cemetery because many individuals died as a result of an inability to fight infection [42], the adult profiles show that some of this workhouse population, who represent the poorest members of society at this time, appear to have suffered from earlier periods of famine which have been recorded in dentine formed during their childhood. The dietary history of Ireland since 1500 as documented by Clarkson and Crawford [3] included many periods of food shortage whether arising from conflicts, extreme weather conditions, or political mismanagement. It was always those with the least entitlement to food who suffered the most [43]. It is possible, therefore, that older individuals in the Kilkenny cemetery had survived earlier periods of food shortages in the half century preceding their death in the Kilkenny Union workhouse. KUW 5, an adult female whose teeth would have been forming during the 1827 famine, shows both patterns of opposing co-variance in her dentine profile (Fig 6): the maize pattern between the ages of 4 and 7 years, when the δ^13^C values temporarily rise above -20‰ coupled with a 1.4‰ fall in δ^15^N, followed by the famine pattern where a 1.1‰ fall in δ^13^C values to within the C_3_ range is coupled with a 1.6‰ rise in δ^15^N denoting a further nutritional stress event between the ages of 8.5 and 12 years. The later-life values of the rib suggest both δ^13^C and δ^15^N are raised above those expected for a nutritionally sufficient potato-dominated C_3_ diet in the years before death.

## Conclusion

This study is the first to use the stable isotope analysis of nitrogen and carbon in human dentine to identify changes related to a well-documented historical period of famine. These data are a poignant reminder of the extreme conditions suffered by the children buried at Kilkenny, but also clearly demonstrate that the intervention provided by the introduction of maize allowed many to recover: their nitrogen isotope ratios return to dietary rather than stress levels, and they survived beyond the dietary change. The ability of the human body to adapt to nutritional deprivation and survive, returning to homeostasis, is clear within these profiles.

Although the change in diet from a C_3_ to a C_4_-based diet is recorded in the isotope ratios of the adult rib bone collagen, it is difficult to resolve the age at which this occurred because of averaging as a result of bone turnover. Using evidence from the dentine collagen δ^13^C and δ^15^N profiles and the historical records for both the importation of maize and the timing of cemetery closure we have been able to identify more precisely when in their lifetime the children from this study who died during the Famine period received relief food. The raised δ^15^N and lower δ^13^C (famine pattern of opposing covariance) in the dentine profiles of the children is marked by the return to lower δ^15^N values and higher δ^13^C in the maize pattern of opposing covariance. Short-term δ^13^C and δ^15^N changes in dentine resulting from physiological, in this case specifically nutritional, stress has not previously been demonstrated in an historical population where the records of the period of famine can be related to individuals who suffered. Further work needs to be undertaken to refine the ageing of each increment, both chronological age and the period of time represented, and whether partially mineralised teeth need ageing differently to complete ones. Nonetheless, this study shows that using incremental dentine collagen δ^13^C and δ^15^N analysis it is possible to identify periods of physiological stress such as famine in both adult and juvenile skeletons if it occurred during tooth development, i.e. up to approximately 23 years of age. This could have important forensic and archaeological applications for the identification of populations and individuals for whom nutritional stress may have contributed to their death.

## Supporting Information

S1 Fig Incremental dentine carbon (δ^13^C) and nitrogen (δ^15^N) isotope ratio profiles for KUW 2, 3, 6, 7, 8, and 9 show that their childhood δ^13^C values reflect a C_3_ potato-based predominantly range from -21‰ and -20‰ Analytical uncertainty is shown at +/- 0.4‰ (2sd). (TIF)

S2 Fig Incremental dentine carbon (δ^13^C) and nitrogen (δ^15^N) isotope ratio profiles for KUW 15, 17, 18, 19 and 20 show that their childhood δ^13^C values reflect a C_3_ potato-based predominantly range from -21‰ and -20‰ Analytical uncertainty is shown at +/- 0.4‰ (2sd). (TIF)

S3 Fig Incremental dentine carbon (δ^13^C) and nitrogen (δ^15^N) isotope ratio profiles for KUW 11, 13, 14 and 16 showing that where there has been a C_3_ to C_4_ dietary change prior to death, rib and final dentine increments can record different δ^13^C values. Analytical uncertainty is shown at +/- 0.4‰ (2sd). (TIF)
