# Supplementary figures and images for: The Great Irish Famine: Identifying Starvation in the Tissues of Victims Using Stable Isotope Analysis of Bone and Incremental Dentine Collagen
Source: PLoS One. 2016 Aug 10;11(8):e0160065. doi: 10.1371/journal.pone.0160065 (PMC4980051; doi:10.1371/journal.pone.0160065)

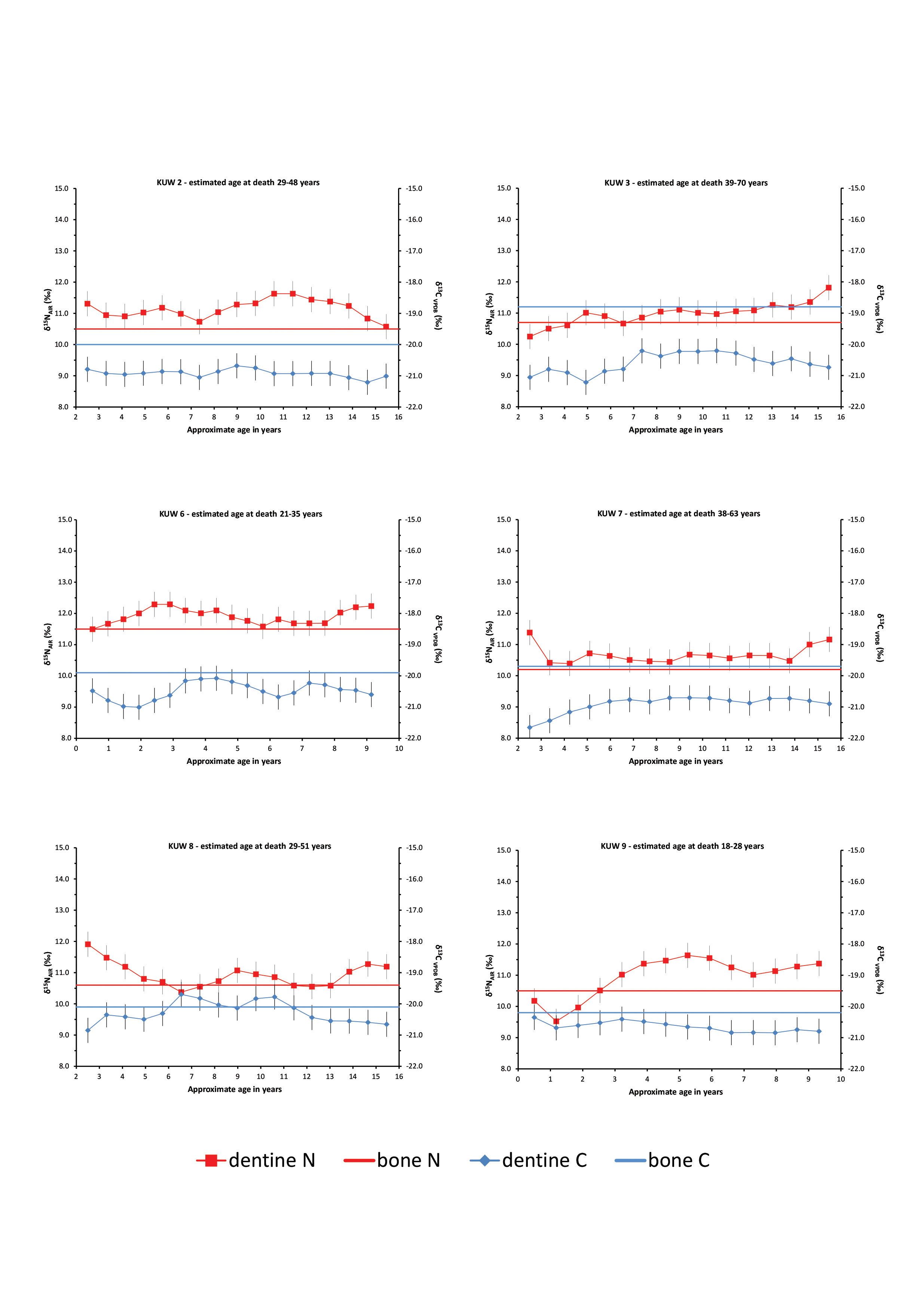

Supplement: S1 Fig — (TIF) [file pone.0160065.s001.tif]

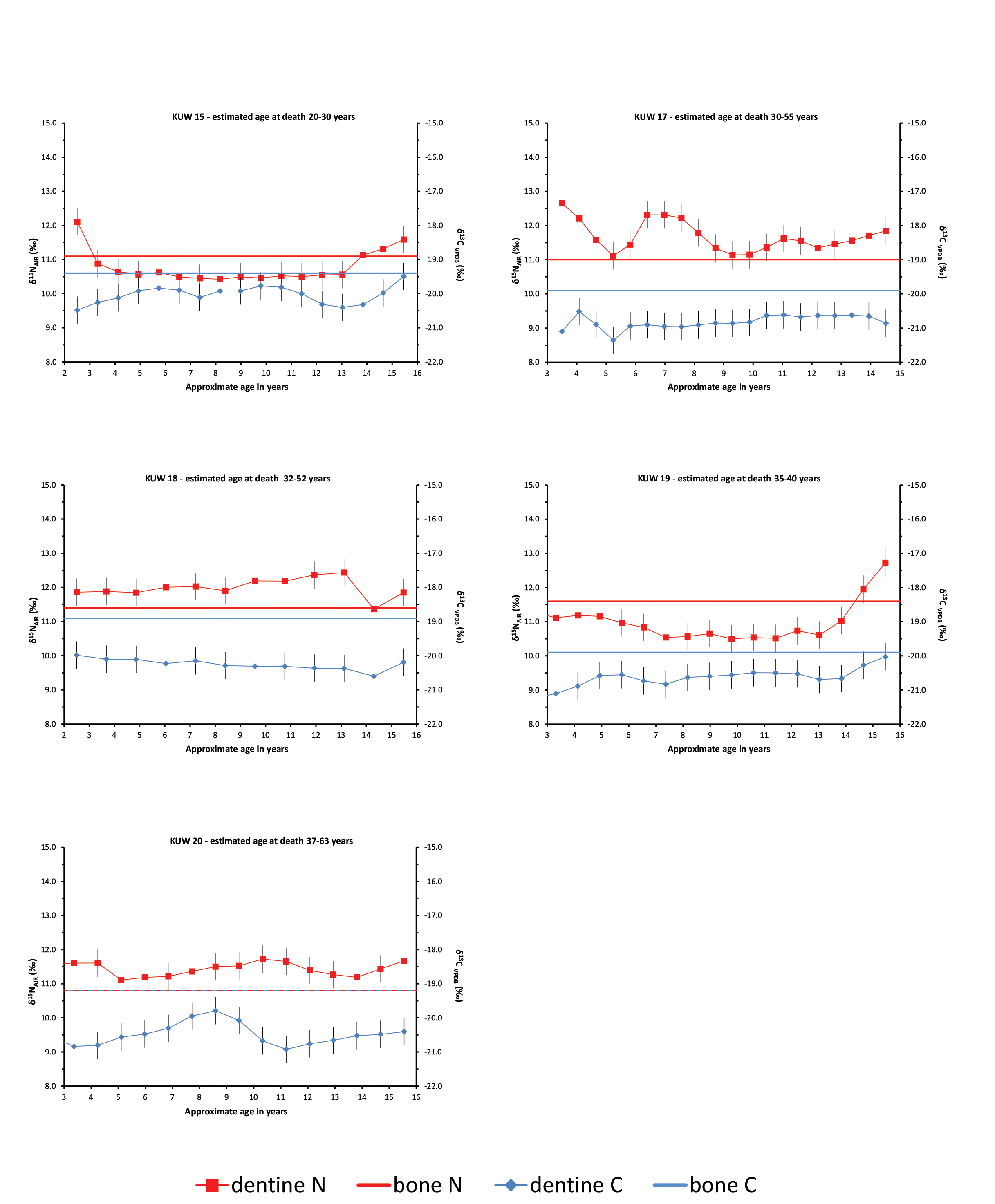

Supplement: S2 Fig — (TIF) [file pone.0160065.s002.tif]

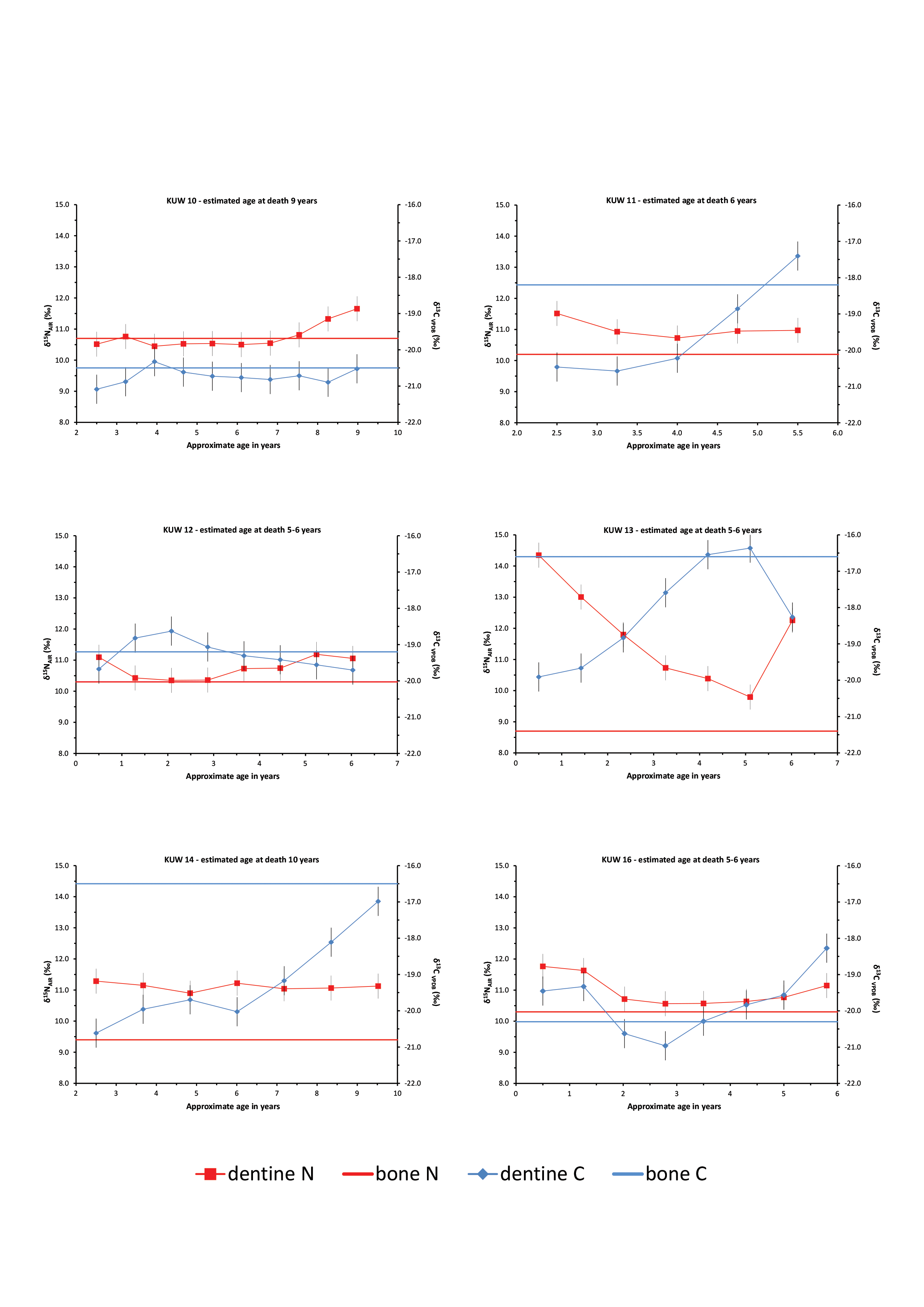

Supplement: S3 Fig — (TIF) [file pone.0160065.s003.tif]
